# Efficacy of a Therapeutic Diet on Dogs With Signs of Cognitive Dysfunction Syndrome (CDS): A Prospective Double Blinded Placebo Controlled Clinical Study

**DOI:** 10.3389/fnut.2018.00127

**Published:** 2018-12-12

**Authors:** Yuanlong Pan, Gary Landsberg, Isabelle Mougeot, Stephanie Kelly, Hui Xu, Sandeep Bhatnagar, Cari L. Gardner, Norton W. Milgram

**Affiliations:** ^1^Nestlé Purina Research, St. Louis, MO, United States; ^2^CanCog Technologies, Toronto, ON, Canada; ^3^Vivocore Inc., Toronto, ON, Canada

**Keywords:** dog, antioxidants, arginine, B vitamins, BPB, cognitive dysfunction syndrome, medium-chain triglycerides, omega-3 PUFAs

## Abstract

Cognitive dysfunction syndrome (CDS) is a common condition in senior dogs, which may be analogous to dementia such as Alzheimer's disease (AD) in people. In humans, AD has been associated with many risk factors such as reduced cerebral glucose metabolism, docosahexaenoic acid (DHA) deficiency, chronic oxidative stress, and chronic inflammation. By targeting some of these risk factors, we have developed two nutritional solutions (medium chain triglyceride, MCT and Brain Protection Blend, BPB) to enhance cognitive function and slow aging-induced cognitive decline. These have been positively evaluated in colony housed senior dogs and cats. The objective of this clinical study was to evaluate the effects of diets with MCTs and the BPB on client-owned dogs with CDS. Participating veterinary clinics screened senior dogs for signs of CDS as determined by a Senior Canine Behavior Questionnaire and a Canine Medical Health Questionnaire. Eighty-seven dogs were randomly enrolled into one of three diet groups with 29 dogs per group: Control, 6.5% MCT oil + BPB (6.5% MCT diet), 9% MCT oil + BPB (9% MCT diet). Diets were fed for a period of 90 days, and each dog's CDS signs were re-evaluated at day 30 and day 90. All 6 categories of the CDS signs were significantly improved (*p* <0.05) in the dogs given the 6.5% MCT diet at the end of the 90-day study. Control only improved in 4 out 6 categories. The 9% MCT diet only improved in dogs that accepted the diet. The results from this dog study confirm the benefits of MCT and BPB in managing clinical signs of CDS in dogs. The results support our hypothesis that targeting known risk factors associated with brain aging and AD is able to improve symptoms of CDS in dogs. These data may facilitate the development of similar nutrient blends to manage MCI and AD.

## Introduction

Cognitive dysfunction syndrome (CDS) is a major condition affecting senior dogs (1, 2) that has parallels to human dementia. Both CDS in dogs and Alzheimer's disease (AD) in humans share similar neuropathological changes including severe cortical atrophy, cerebral amyloid angiopathy and ventricular enlargement (3–5). Multiple risk factors have been associated with accelerated brain aging and higher risk of AD, including DHA deficiency (6), elevated blood homocysteine (7), low status of vitamin B6, vitamin B12, and folic acid (8), high blood pressure (9), chronic oxidative stress (10), and chronic low grade inflammation (11). Since it is impossible to address the above-mentioned multiple risk factors with single nutrient, we have selected a blend of nutrients (Brain Protection Blend, BPB) which included enhanced levels of B vitamins, antioxidants, omega-3 fatty acids and arginine based on their ability to prevent or reduce these risk factors. We evaluated the effects of BPB on cognitive function in healthy middle aged and senior cats, and senior dogs with established cognitive test protocols including landmark, egocentric and delayed-none-matching-to-position tests, and the results confirmed that the BPB was able to enhance cognitive function and retard aging-induced decline in learning, memory, and executive function (12, 13). A recent study showed that in human subjects with mild cognitive impairment, dietary supplementation of B vitamins was able to reduce cognitive decline only in subjects with high levels of blood omega-3 fatty acids (14).

Another common change associated with aging is the brain's ability to metabolize glucose. Cerebral glucose metabolism is reduced in healthy old people (15) and healthy old animals including rodents (16), dogs (17), and monkeys (18). Alexander et al. (19) reported that cerebral glucose metabolism was significantly lower in old patients with Alzheimer's disease (AD) than in age-matched healthy old subjects, which confirmed a previous report that cerebral glucose utilization has been significantly reduced in AD subjects (20). MCT supplementation or exercise improved memory performance in AD patients by providing the brain with ketone bodies as an alternative energy source (21, 22). Our study showed that dietary MCT was able to improve learning, memory and executive function in senior dogs (23). These data indicate that ketone bodies may play an important role in supporting brain function by serving as an energy source in both humans and dogs.

MCT and BPB enhance cognitive function with different mechanisms of action, therefore we hypothesized that a combination of MCT and BPB would deliver synergistic beneficial effects on cognitive function in dogs with CDS. Since cerebral glucose metabolism is significantly lower in old AD patients compared to age-matched healthy old subjects (19), we increased dietary MCT from the 5.5% level to 6.5% or 9% so that more energy could be available to compensate for the marked reduction in cerebral glucose metabolism in dogs with CDS.

Dogs with CDS present with clinical signs in a number of behavioral domains including disorientation, altered social interactions, sleep/wake disturbances, house soiling, anxiety and activity, which may be referred to by the acronym DISHAA (1, 2, 24–34). Therefore, for this clinical study we used a questionnaire (35) that incorporated all 6 DISHAA categories (Supplementary Table 1), including questions from all previously validated questionnaires to have a complete and sensitive screening tool for identifying dogs that had levels ranging from mild to severe (27, 36–39).

The objective of this clinical study was to evaluate the effects of combinations of 6.5% MCT and BPB (6.5% MCT diet) or 9% MCT and BPB (9% MCT diet) on signs of CDS in senior dogs.

## Experimental Methods

### Study Design

The study was a randomized, double-blind, placebo-controlled, multi-site study carried out at 24 different veterinary clinics within the province of Ontario. The study protocol was approved by the CanCog Technologies Institutional Animal Care Committee (VRI5-15142-CC), and followed the guidelines of the Ontario Ministry of Agriculture. Each pet owner was required to sign an informed and written consent form prior to starting his or her pet on the trial. Eligible dogs were >9 years of age and exhibited signs associated with CDS. Upon enrollment, dogs were randomly allocated to one of the following three groups of 29 dogs per group: Control diet, 6.5% MCT diet, and 9% MCT diet.

At the pre-screening visit on day-8, a physical examination, blood work and urinalysis were performed in addition to completion of a Senior Canine Behavior Questionnaire and a Canine Medical Health Questionnaire (Supplementary Tables 1, 2), to confirm eligibility of the subject. Following group placement, appropriate food was distributed to pet owners and feeding of the assigned diet continued for 90 days.

After completion of a mid-study (Day 30) cognitive and behavioral assessment using the questionnaire, a Day 90 follow up visit included repeat physical examinations, questionnaire, blood work and urinalysis.

### Dog Recruitment

One hundred dogs >9 years of age were screened for the study. Of these, 13 were excluded from the study for abnormal findings, including 1 dog with moderately elevated alanine aminotransferase (ALT) and serum alkaline phosphatase (SAP) that was also deaf; 3 dogs with renal disease; 2 dogs with persistent urinary tract infections; 1 dog with hypercalcemia; 5 dogs with markedly elevated SAP (1,500–4,000 U/L); and one dog with both renal disease and moderately elevated ALT. Further diagnostics were not performed within the study.

Eighty-seven dogs (48 males and 39 females) of various breeds (52 different breeds) were enrolled with 29 animals assigned to each treatment group. The ages of dogs ranged from 9 to 16 years. Dogs were obtained from individual dog owners with signed consent and were either intact or neutered.

Participating veterinary clinics performed pre-screening visit procedures on potential dogs which were senior dogs with canine cognitive dysfunction syndrome as determined by a Senior Canine Behavior Questionnaire (Supplementary Table 1) and a Canine Medical Health Questionnaire (Supplementary Table 2). The Study Coordinator in conjunction with the Principal Investigator determined if the subject was suitable for enrollment based on the Owner Informed Consent Form, physical examination including body weight and body condition score, blood results for CBC, clinical chemistry, urinalysis, and the Senior Canine Behavior Questionnaire and the Canine Medical Health Questionnaire.

For inclusion, dogs had to be 9 years or older, reside in their current home for at least 24 months, and have a positive CDS response on at least two of the categories on the Senior Canine Behavior Questionnaire. Dogs treated with a calming product or cognitive enhancing product within the previous 4 weeks could be included if they were maintained on the product for the duration of the study. Dogs that were on a diet meant to treat cognitive decline were required to be off the diet for at least 30 days prior to enrollment.

Dogs were excluded if they had medical problems that may have caused the behavioral signs, including dogs with marked hearing loss, marked visual deficits, moderate to marked osteoarthritis, or if medical problems precluded a change in diet.

### Group Assignment

Cases that met inclusion criteria for enrollment were assigned to one of three diet groups according to a random number generated by randomization schedule. The three diet types were provided in identical packaging and were pre-labeled as A, B, or C to ensure double blinding. Twenty-nine dogs were assigned to each group.

Dogs treated with a calming product or cognitive enhancing medication for more than 4 weeks prior to study initiation were spread evenly across groups to the extent possible. Of 10 dogs that were being treated with these products, three dogs including one on clomipramine, one on essential fatty acids (withdrew), and one amitriptyline (withdrew) were assigned to diet A, three dogs including two on an omega-3 supplement (both withdrew) and one on an l-theanine, colostrum, valerian supplement were assigned to Diet B and four dogs, two on gabapentin, and two on omega-3 supplements were assigned to Diet C.

When multiple dogs from one household were enrolled in the trial, all dogs in the home were assigned to the same group in order to avoid cross contamination of the study diets. This occurred for 2 pairs of dogs in 2 households assigned to diet C, 2 dogs in the same household assigned to diet B, and 4 dogs from the same household in diet A.

### Control and Test Diets

The control diet was an experimental diet for adult dogs with all the essential nutrients exceeding the minimal nutrient requirements for canine adult maintenance defined by the Association of American Feed Control Officials (AAFCO). The 6.5% MCT diet was formulated by replacing 6.5% tallow with 6.5% MCT oil. The 9% MCT diet was formulated by adding 2.5% more MCT oil to the 6.5% MCT diet by reducing carbohydrate content by 2.5%. All diets were manufactured by Nestle Purina PetCare, Inc., (Saint Louis, Missouri, USA), and contained the same levels of protein, crude fiber and moisture. Dietary ingredient and chemical composition are provided in Table 1. Diet samples were sent to Nestle Purina Analytical Laboratories (Nestle Purina PetCare, St. Louis, Missouri, USA) for chemical analyses. Ash, crude fat, crude fiber, crude protein, moisture, fatty acid profile (linoleic acid, capric acid, caprylic acid) were measured based on Association of Official Agricultural Chemists (AOAC) Methods 942.05, 922.06, 962.09, 990.03, 930.15, and 996.06, respectively.

**Table 1:** Ingredients and chemical composition of diets as fed.

	**Control**	**6.5% MCT diet**	**9% MCT diet**
Crude Protein (%)	32.9	32.5	32.9
Crude Fat (%)	17.9	18.2	21.0
Moisture (%)	6.6	6.8	6.9
Crude Fiber (%)	1.70	1.57	1.27
Ash (%)	6.5	6.3	6.5
MCT (%)^*^	0	6.5	9
**BPB INGREDIENTS**			
DHA (%)	0	0.23	0.22
EPA (%)	0	0.30	0.29
Arginine (%)	1.78	1.79	1.93
Thiamine (B1, mg/kg)^*^	8.16	58.68	56.57
Riboflavin (B2, mg/kg)	5.60	26.5	28.3
Niacin (B3, mg/kg)^*^	86.99	225.76	220.78
Pantothenic acid (B5, mg/kg)	19.20	77.3	83.2
Pyridoxine (B6, mg/kg)	4.39	17.8	18.5
Folic acid (B9, mg/kg)	1.64	8.39	9.32
Cobalamine(B12, mg/kg)	0.076	0.175	0.156
Vitamin E (IU/kg)	52.6	552.08	490.44
Vitamin C (mg/kg)	9.65	151	143
Selenium (mg/kg)	0.704	0.681	0.806
Calculated ME^**^ (KJ/g)	15.67	15.66	16.26
Main ingredients	Chicken, Rice, Corn Gluten Meal, Poultry By-Product Meal, Dried Egg, Wheat Flour. Medium Chain Triglyceride, Fish Meal, Soy Protein Isolate, Tallow Edible w/Vitamin E.		

* *Based on formulation values. All other nutrients were the analytical values immediate after production*.

** *Calculated based on the predictive equation for metabolizable energy (ME) in dog foods (40)*.

Both control and test diets had comparable macro and micronutrient profiles except MCT levels and higher levels of BPB nutrients (DHA, EPA, vitamin E, vitamin C, and B vitamins). Ascorbyl-2-polyphosphate was used as the form of vitamin C to ensure the stability of vitamin C in the test diet during the clinical study. Arginine level was increased over control diet only in the 9% MCT diet, but the arginine content in the control and 6.5% MCT diets was three times higher than the AAFCO minimum maintenance requirement (Table 1). In addition, fat level was increased to 21% in the 9% MCT diet due to the addition of higher level of MCT oil. Since many of the BPB nutrients are components of the ingredients of the diet, most of the BPB nutrients were several times over the AAFCO maintenance requirements for dogs except DHA, EPA, vitamin C, and vitamin E. In addition, Vitamin E in the control diet met the AAFCO maintenance requirement. Higher levels of protein provided the natural source of arginine so the levels were similar for control and 6.5% MCT diet. Therefore, it was possible that the control diet also had positive effects on the brain health in dogs.

### Administration of Test and Control Diets

After enrollment approval, bags of the assigned diet were shipped to the corresponding veterinary clinic for pickup by the pet owners. The assigned diet was to serve as the sole feed for a period of 3 months (90 days). Feeding guidelines were included with each diet and dogs were fed to maintain their body weight. Owners were instructed to maintain the feeding regimen prior to the trial. Assigned diets were introduced gradually over a 7-days period.

### Health Observations and Physical Examinations

Dog health observations were performed daily over the course of the study by the dog owner. Any serious, prolonged, and/or life threatening health observations were to be reported to the participating veterinarian and Principal Investigator to determine if removal from study was warranted.

Physical examinations were carried out by the site veterinarian according to industry standard and included an assessment of all body systems. Exams occurred at baseline (study Day-8) and again at study conclusion (Day 90 ± 10 days).

### Behavioral and Cognitive Assessment

The pet owner was required to complete a Senior Canine Behavior Questionnaire (Supplementary Table 1) and a Canine Medical Health Questionnaire (Supplementary Table 2), which assessed cognitive function, and behavioral health status. The questionnaire was completed as part of the initial baseline visit, on Day 30 (±5 days) and again on Day 90 (±10 days). Scoring scale of the questionnaire for the CDS signs had four scores (0 = none or no change, 1 = mild, 2 = moderate, 3 = severe). The maximal scores for DISHAA are 21, 21, 15, 18, 21, and 18, respectively. Scores for enrolled dogs ranged from 6 to 85. Of the 74 dogs that completed the trial, 41 dogs had scores <30 indicating a milder dysfunction and 33 had scores ≥30 indicating a more severe dysfunction.

### Blood Sample Collection

At the time of the initial veterinary examination and again on Day 90 (±10 days), a blood sample was collected from each animal for the purpose of complete blood count, clinical chemistry and β-hydroxybutyrate (BHB) analysis. Approximately 10 ml of whole blood was collected from a suitable vein. Of the 10 ml collected, 2 ml was placed into a K_2_EDTA tube, and 8 ml was placed into two separated 4 ml serum separator tubes. Tubes were refrigerated until sent to Antech Diagnostics for analyses.

### Urine Sample Collection

A urine sample was collected from each dog during the Day-8 visit and again on Day 90 (±10 days) for the purpose of urinalysis. If a sample could not be obtained at the initial visit, it was required within 7 days for the animal to be considered for enrollment. A minimum of 5 ml of urine was collected according to industry standard practices by natural voiding, cystocentesis, or catheterization. Samples were placed in a sterile container and refrigerated until sent to Antech Diagnostics for analysis.

### Blinding

The study was double-blinded to the pet owner, study veterinarian, study coordinator, and clinical laboratory staff. Therefore, all people collecting data were blinded to the treatment of each dog.

### Statistical Analysis

Statistical analyses were carried out using StatSoft, Inc. (2012). STATISTICA (data analysis software system), version 11, www.statsoft.com. Each subtest of the questionnaire data was initially analyzed using a repeated measures ANOVA with Group as between subject variable and age at test (baseline, 30 and 90 days) as a within subject variable. Although none of the analyses revealed statistically significant age by treatment interaction, the analyses did yield significant age at test effects. In instances where there were significant age-at-test effects, we used the Fisher Exact Probability test for multiple comparisons to determine which of the differences achieved statistical significance. The sample size was determined by the feasibility of recruitment.

One dog in group B (Case 67) was removed from the data analysis, as the dog was enrolled in error as it had been fed a diet (Royal Canin Mature Consult) that was on the exclusion list.

For the analysis of blood chemical and CBC data, a linear mixed effects model was run where ID was entered in as a random effect and Time (initial/baseline vs. final/90 days), Diet (A, B, and C), and Time^*^Diet were entered as fixed effects. A mixed effect model was run because it allows for control for the repeated nature of the data. LS means and differences in LS means are also provided.

A retrospective power analysis was conducted to determine the sample size for each of the six subsets of CDS signs within each dietary group, using the data collected from this study and a power of 0.80 and a significance level of 0.05. The results showed that the sample sizes for the control group, 6.5% MCT diet group and 9% MCT diet group ranged from 17 to 232, 7 to 30, and 31 to 372, respectively, depending on the subset differences between the baseline and end of the study. These data indicated that we had adequate power mostly in the 6.5% MCT diet group.

## Results

### Effects of the Test Diets on Clinical Signs of Dogs With CDS

**Figure 1:**
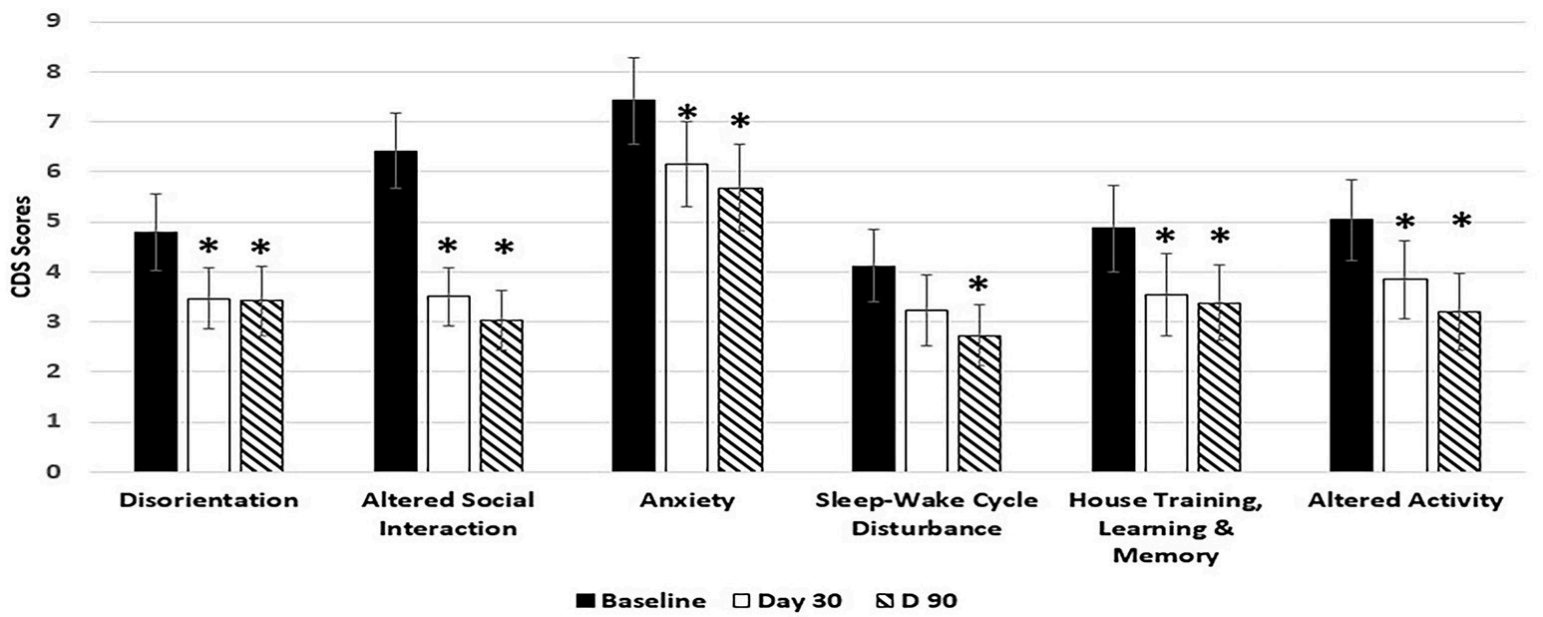
Effects of 6.5% MCT diet on CDS signs at day 30 and day 90. Twenty-nine client owned dogs were assessed for six different behavioral signs of cognitive dysfunction syndrome (CDS) and then placed on the 6.5% MCT diet. The dogs were re-assessed after 30 days and again after 90 days. The bars illustrate Means ± SEM for each of the measures at baseline, 30 days and 90 days. Fisher Exact Probability test for multiple comparisons was used to compare baseline with day 30 and day 90. The asterisks indicate statistically significant differences (*p* < 0.05) from baseline. Two dogs dropped out of the study, and one dog was removed from the study due to enrollment error.

Dogs fed the 6.5% MCT diet showed significant improvement over baseline in all 6 categories of CDS signs including three categories related to brain cognitive functions (disorientation, altered social interaction, and loss of house training) at day 90, and most of the improvements (5 out of the 6 categories) were observed as early as day 30 during the study (Figure 1). Of the dogs on 6.5% MCT diet that completed the study, 23/26 dogs were responders (improvement or no further progression) and 3 were worse. The greatest number of dogs improved in the category of social interactions in which 24/26 dogs were responders (improvement or no further progression) and 2 worse. For disorientation, 19 dogs were responders and 4 dogs were worse. Comparing dogs with low and high CDS scores, for dogs with CDS scores <30, 10 dogs improved, 2 worsened and 1 had not change, while for dogs with CDS scores 30 and over, 11 dogs improved, 1 worsened, and 1 was the same.

**Figure 2:**
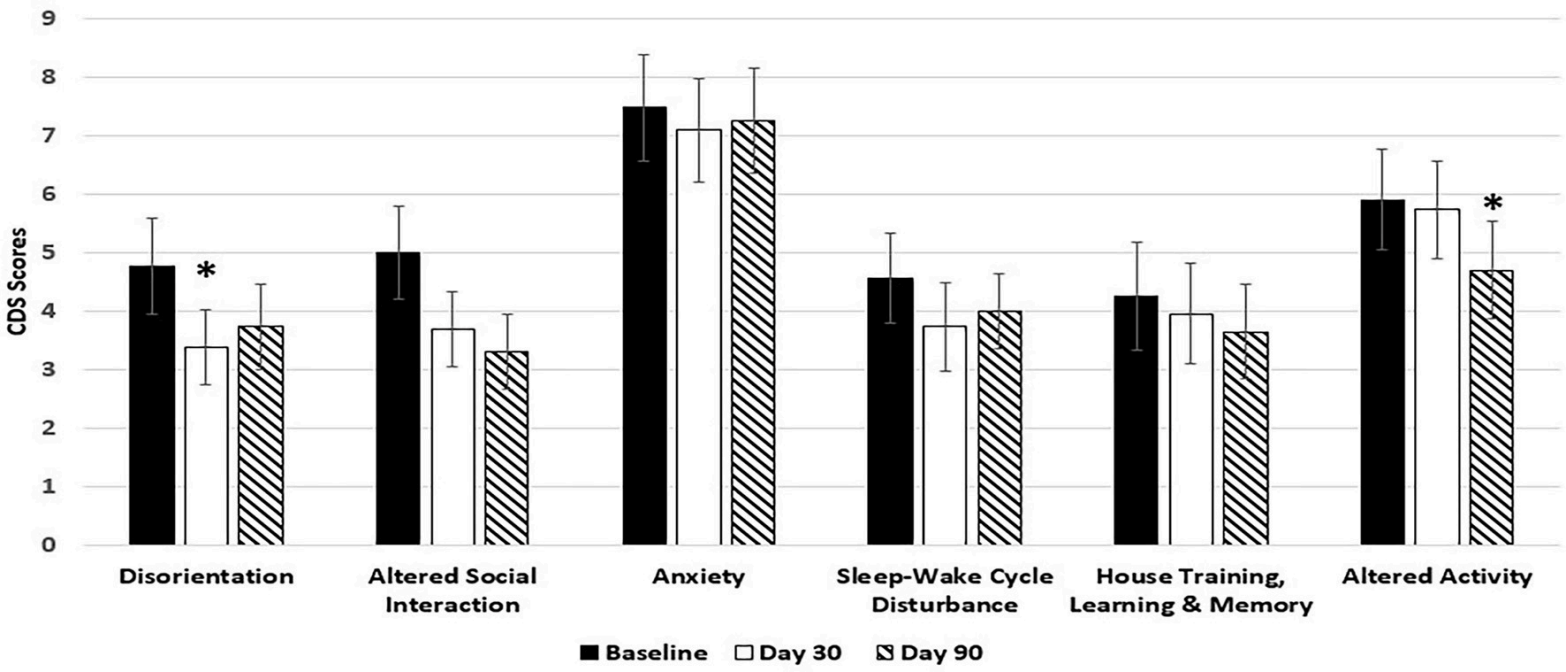
Effects of 9% MCT diet on CDS signs at day 30 and day 90. Twenty-nine client owned dogs were assessed for six different behavioral signs of cognitive dysfunction syndrome (CDS) and then placed on the 9% MCT diet. The dogs were re-assessed after 30 days and again after 90 days. The bars illustrate Means ± SEM for each of the measures at baseline, 30 days and 90 days. Fisher Exact Probability test for multiple comparisons was used to compare baseline with day 30 and day 90. The asterisks indicate statistically significant differences (*p* < 0.05) from baseline. Six dogs dropped out of the study.

**Figure 3:**
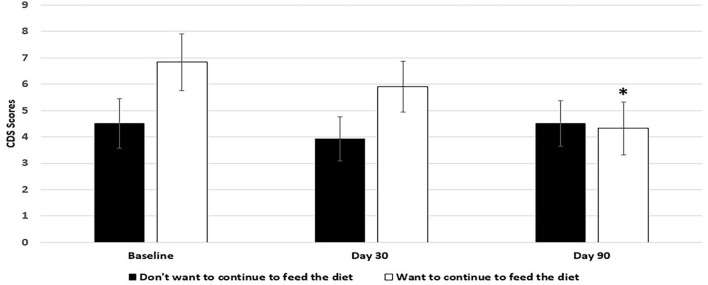
Effects of owners' comments on 9% MCT diet on the outcome of the diet. Twenty-nine client owned dogs were assessed for six different behavioral signs of cognitive dysfunction syndrome (CDS) and then placed on the 9% MCT diet. The dogs were re-assessed after 30 days and again after 90 days. The bars illustrate Means ± SEM for the mean score of six different behavioral signs at baseline, 30 days and 90 days. Six dogs dropped out of the study. Out of the 23 dogs remaining at the end of the study, 12 dog owners answered that they do not want to continue to feed their dogs with the diet, and 9 dog owners answered that they want to continue to feed their dogs with the diet. Fisher LSD test was used to analyze the data between the baseline and day 30 or baseline and day 90. The asterisks indicate statistically significant differences (*p* < 0.05) from baseline.

Dogs fed the 9% MCT diet failed to significantly improve in most of the signs over baseline because of smaller sample size due to larger dropout rate. However, the signs in 5 out of 6 categories were numerically reduced in the dogs fed 9% MCT diet (Figure 2). Further analysis showed that dog owners' attitudes about the 9% MCT diet determined the outcome of this group. Dogs from owners who would not continue to feed their dogs the diet after the clinical study did not show significant improvement at the end of the study. On the contrary, dogs from owners who would continue to feed their dogs the diet after the clinical study showed significant improvements at the end of the study (Figure 3).

### Effect of Control Diet on Clinical Signs of Dogs With CDS

**Figure 4:**
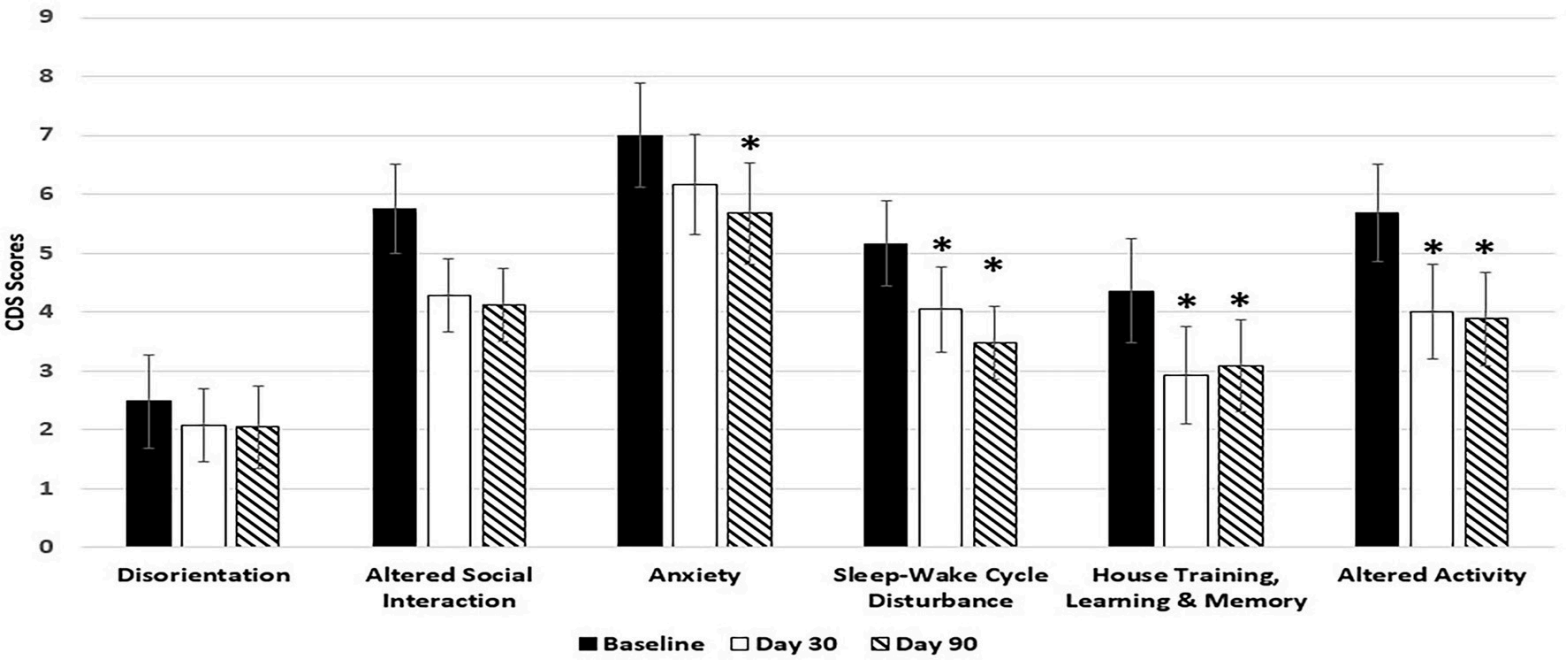
Effects of Control diet on CDS signs at day 30 and day 90. Twenty-nine client owned dogs were assessed for six different behavioral signs of cognitive dysfunction syndrome (CDS) and then placed on the control diet. The dogs were re-assessed after 30 days and again after 90 days. The bars illustrate Means ± SEM for each of the measures at baseline, 30 days and 90 days. Fisher Exact Probability test for multiple comparisons was used to compare baseline with day 30 and day 90. The asterisks indicate statistically significant differences (*p* < 0.05) from baseline. Four dogs dropped out of the study.

At day 30, control diet significantly improved 3 (Sleep-wake cycle, house training and altered activity) out of 6 categories of CDS symptoms. Dogs in the control group showed significant improvement in 4 out of the 6 categories of CDS symptoms at day 90. But the control diet failed to significantly improve two out of the three categories of CDS symptoms related to brain cognitive function (disorientation, and altered social interaction) (Figure 4).

### Effects of the Diets on Blood Lipid Profiles

Both test diets significantly increased blood levels of DHA, EPA, DHA, and EPA, total omega-3 PUFAs, and omega-3/omega-6 ratios compared with the control diet (Table 2). However, even though both test diets had the same inclusion rate of DHA and EPA (Table 1), dogs fed the 9% MCT diet had significantly lower blood EPA, DHA, and EPA, total omega-3 PUFAs, and omega-3/omega-6 ratio than the dogs fed the 6.5% MCT diet (Table 2). These data indicated that pet owners might have mixed the 9% diet with other diets, which diluted the intake of DHA and EPA.

**Table 2:** Effects of the diets on blood omage-3 and omega-6 levels.

**Diets**	**DHA (%)**	**EPA (%)**	**DHA+EPA (%)**	**AA (%)**	**Total N-3 PUFAs (%)**	**Total N-6 PUFAs (%)**	**N-3/N6 ratio**
Control (*n* = 12)	1.03 ± 0.17	0.49 ± 0.12	1.53 ± 0.17	16.93 ± 1.74	3.09 ± 0.43	42.79 ± 2.22	0.071 ± 0.01
6.5% MCT diet (*n* = 9)	3.00 ± 0.36^*^	3.31 ± 0.42^*^^#^	6.31 ± 0.76^*^^#^	14.70 ± 0.61	8.85 ± 0.86^*^^#^	42.23 ± 1.06	0.212 ± 0.02^*^^#^
9% MCT diet (*n* = 11)	2.07 ± 0.33^*^	1.73 ± 0.33^*^^#^	3.81 ± 0.63^*^^#^	14.12 ± 1.35	5.80 ± 0.80^*^^#^	38.77 ± 1.86	0.146 ± 0.02^*^^#^

* *Significantly (p < 0.05) different from control*.

# *Significantly (p < 0.05) different between 6.5% MCTs and 9% MCT diets*.

### Effects of the Diets on Complete Blood Count (CBC)

All the CBC parameters were within the normal range for adult dogs (data not shown). No significant differences were observed at both baseline and end of the study between control and either of the two test groups in WBC, RBC, hemoglobin, hematocrit, platelet count, neutrophils, lymphocytes, monocytes, eosinophils, and MCH (data not shown). MCV in control dogs was significantly higher than the 9% MCT group at the end of the study (71.71 ± 0.89 vs. 68.96 ± 0.91), and MCHC was significantly lower in the control dogs than in dogs from both test groups at the end of the study (326.96 ± 3.21 vs. 336.35 ± 3.10 or 337.59 ± 3.35).

### Effects of the Diets on Blood Chemical Parameters

All the blood chemical parameters were within the normal range (data not shown). No significant differences at both baseline and end of the study between control and either of the two test groups was observed for globulin, A:G ratio, alkaline phosphatase, total bilirubin, glucose (Table 3), calcium, sodium, potassium, sodium:potassium ratio, chloride, bicarbonate, cholesterol (Table 3), triglycerides (Table 3), and fasting BHB (Table 3). Total protein and albumin differed between control and the 6.5% MCT diet at the end of the study (68.24 ± 0.93 vs. 64.71 ± 0.88, 37.66 ± 0.76 vs. 35.44 ± 0.73, respectively). Serum ALT and BUN differed between control and 6.5% MCT diet at baseline (58.14 ± 5.31 vs. 41.80 ± 5.49, 5.48 ± 0.37 vs. 6.76 ± 0.36, respectively). Additionally, creatinine, and anion gap differed between the control and 6.5% MCT diet at the end of the study (74.21 ± 3.96 vs. 88.35 ± 3.91, 21.38 ± 0.60 vs. 19.45 ± 0.64, respectively). Finally, both cholesterol and BHB were significantly higher at the end of the study than the baseline in dogs fed the 9% MCT diet.

**Table 3:** Effects of the diets on fasting blood glucose, BHB, cholesterol, and triglycerides in dogs.

	**Glucose (mmol/l)**			**Beta-hydroxybutyrate (μmol/l)**		
Control	5.16 ± 0.14	5.07 ± 0.14	0.600	82.76 ± 8.20	78.72 ± 8.20	0.686
6.5% MCT	5.12 ± 0.14	5.26 ± 0.14	0.400	74.50 ± 8.05	61.96 ± 8.05	0.204
9% MCT	4.91 ± 0.17	5.11 ± 0.14	0.300	76.43 ± 8.55	97.43 ± 8.55	0.047
	**Cholesterol (mmol/l)**			**Triglycerides (mmol/l)**		
	**Baseline**	**End of Study**	***P*****-value**	**Baseline**	**End of Study**	***P*****-value**
Control	6.71 ± 0.31	6.86 ± 0.31	0.500	1.53 ± 0.16	1.56 ± 0.16	0.900
6.5% MCT	6.57 ± 0.30	6.57 ± 0.30	0.999	1.48 ± 0.15	1.30 ± 0.16	0.300
9% MCT	6.82 ± 0.31	7.75 ± 0.33	0.0002	1.38 ± 0.18	1.43 ± 0.19	0.800

### Drop-Out

At the end of the study, four dogs in the control group, 2 dogs in the 6.5% MCT diet group and 6 dogs in the 9% MCT diet group failed to complete the study. At 30 days 2 dogs in the 9% MCT diet dropped out, one due to owner non-compliance and one due to an abdominal tumor, while at 90 days 4 more dogs were dropped due to owner non-compliance, gastrointestinal upset, disinterest in the diet and vestibular disease. In the control group at 30 days, 3 dogs were dropped, one due to owner non-compliance, one due to illness and one that could not transition to diet, and 1 dog was dropped at 90 days due to owner non-compliance. In the 6.5% MCT group at 30 days two dogs had been unable to be transitioned onto the diet.

## Discussion

The main objective of this study was to test the hypothesis that a combination of MCT and BPB will improve clinical signs of dogs with CDS. In this clinical study, we have used a questionnaire including all six categories of CDS symptoms referred to as DISHAA to assess dogs with CDS and evaluate the effects of diet on these CDS signs. The results showed that the 6.5% MCT diet was able to significantly improve five out of the six categories of clinical signs of CDS at day 30 and all six categories of clinical signs of CDS in pet dogs at the end of the 3-months clinical study. This is in comparison to the control diet in which disorientation and social interactions did not improve significantly. As pet owners are particularly sensitive to changes in social interactions, it is perhaps not surprising that they are the most frequently reported signs in mild cognitive decline and amongst the most commonly reported signs (1, 27). Additionally, signs of disorientation are indicative of more advanced stages of dementia and amongst the most common signs in severe CDS (1, 27, 28, 36).

In evaluating individual dogs, 88% improved or did not progress, with an approximately equal number of dogs improving that had high scores ≥30 indicative of more marked dysfunction as those with lower scores <30 (mild dysfunction). Taken together this supports the findings that the diet was effective in improving signs from mild to severe CDS. These results are further supported by the reports showing that in human subjects with mild cognitive impairment, dietary supplementation of B vitamins was able to reduce cognitive decline only in subjects with high levels of blood omega-3 fatty acids (14), and dietary MCT supplementation improved memory performance in AD patients by providing the brain with ketone bodies as an alternative energy source (21).

Higher antioxidants in the BPB of the test diet may be able to further enhance the therapeutic benefit of the diet by attenuating chronic oxidative stress associated with subjects with dementia (10). Higher omega-3 PUFAs of BPB in the test diet resulted in higher blood omega-3 PUFAs which can correct DHA deficiency and lead to better management of chronic low grade inflammation in dementia subjects (11). An optimal level of dietary arginine is able to enhance fasting blood arginine and nitric oxide production in senior dogs, which, in turn, improves blood circulation and cognitive function (13). MCT may potentiate the BPB effects by improving cognitive function through enhanced energy supply to the brain in forms of ketone bodies in dogs with CDS. Therefore, in the current study, the benefits of the test diet containing MCT and BPB on CDS signs come from multiple nutrients working synergistically to enhance cognitive function by eliminating or reducing multiple known risk factors for dementia.

Dogs fed the 9% MCT diet didn't significantly improve most of the signs compared with baseline due to a high drop-out rate (6 out of 29 dogs). Another primary suspected reason for the failure of the 9% MCT test diet to improve CDS signs was poor acceptance of the diet by the dogs, which was confirmed by dog owners' attitudes toward the 9% MCT diet and blood omega-3 PUFA enrichment in the dogs. Even though both test diets had the same inclusion levels of DHA and EPA, dogs fed the 9% MCT diet had significantly lower blood EPA, DHA, and EPA, total omega-3 PUFAs and the ratio of omega-3 and omega-6 PUFAs than the 6.5% MCT diet. These data indicated that some owners did not feed their dog exclusively with the 9% test diet and likely added in other diets, which resulted in lower daily intake of both MCT and BPB nutrients.

Our previous studies have demonstrated that a MCT supplemented diet or the BPB diet was able to enhance cognitive function in healthy senior dogs (13, 22). In those two studies, we used a single breed (Beagle) and established cognition-evaluating protocols to determine whether a MCT diet or BPB diet enhanced learning, memory, and executive function in senior dogs. Responses to the protocols were mostly dependent on dogs' cognitive ability. This clinical study reflected the clinical population with a wide range of breeds and breed mixes. However, previous studies have shown that breeds are not a risk factor for CDS (28, 41). Since it was not possible to use established cognition-evaluating protocols in a clinical study, we used a questionnaire to evaluate any improvement in each of the six CDS signs. The questionnaire was entirely dependent on pet owners' input of their pets' status, which could be subjective. The 6.5% MCT diet significantly improved all three CDS signs (disorientation, altered social interaction, housing training, learning, and memory) related to cognitive function, while the control diet significantly improved only one of these CDS signs. These data suggest that a diet containing both MCT and BPB was able to improve CDS signs by enhancing cognition in dogs with CDS.

The following factors may contribute to the significant effect of the control diet. First, the control diet contained most of the BPB ingredients at levels several times the minimum requirements for adult dogs based on AAFCO profiles, except for DHA, EPA, vitamin C, and vitamin E (Table 1). Therefore, the control diet may have delivered some of the BPB benefits in control-fed dogs. Secondly, a placebo effect is commonly observed in clinical studies and placebo treatments can enhance physical processes of disease more easily and effectively than biochemical processes (42). Because of the potential placebo effects in clinical studies, we initially confirmed the efficacy of MCT and BPB in colony dogs with validated cognition tests, which minimized placebo effects and eliminated any subjective owner evaluation (13, 22). It has been proposed that the placebo effect may be due to a person's expectations of clinical benefits (43). Since all pet owners were blind to the treatments of the study, pet owners in the control group may have thought that their dogs were in the treatment groups, and expected some beneficial changes in their pets.

Since most cases of cognitive dysfunction presenting with mild signs go unreported, and a majority (87%) of cases go undiagnosed, veterinarians must actively screen for behavioral signs at each senior pet visit (27, 41). As 13 of the 100 dogs with signs of cognitive dysfunction in this study were excluded because of underlying medical problems, this also supports the need for blood and urine tests as an essential part of senior pet screening. In fact, considering that 42% of dogs over 9 years of age with no signs of impairment progressed to mild impairment over 6 months (27), health care screening in the senior pet should be scheduled twice a year.

## Conclusion

In summary, the results of this clinical study showed that the 6.5% MCT diet was able to significantly improve all six categories of the CDS signs in dogs with CDS at the end of the 90-day study. Most of the significant benefits in the dogs fed 6.5% MCT diet were observed at day 30. For some dogs, the 9% MCT diet may have had some acceptance issues, diminishing the overall response in dogs with CDS. Dogs that did not have any issues with consumption did show significant improvements in their CDS signs after 90 days. The observed benefits of the control diet may be due to both partial BPB benefits and a placebo effect. The results from this dog study confirm the benefits of MCT and BPB in managing clinical signs of CDS in dogs. The results support our hypothesis that targeting known risk factors associated with brain aging and AD is able to improve symptoms of CDS in dogs. These data provide pre-clinical data to support clinical studies testing similar nutrient blend in subjects with MCI or AD.

## Author Contributions

GL, NM, and YP designed the study, interpreted the results, and prepared the manuscript. GL and IM visited participating clinics and taught veterinarians how to evaluate clinical signs of CDS in dogs. GL, IM, and SK coordinated the clinical study. HX formulated the diets and SB scheduled and supervised the production of the diets. CG, HX, SB, and YP are employees of Nestlé Purina Research and have not been involved in dog recruitments, randomization, data and sample handling, data analysis, and storage except that CG analyzed the data of blood lipid profiles, Complete Blood Count, and blood chemical parameters.

### Conflict of Interest Statement

YP is one of the inventors for EP2194781B1-Compositions and methods for enhancing cognitive function. CG, HX, SB, and YP are employees of Nestlé Purina Research. The authors have no other relevant affiliations or financial involvement with any organization or entity with a financial interest in or financial conflict with the subject matter or materials discussed in the manuscript apart from those disclosed. IM is the President and CEO of CanCog Technologies. NM is the Chief Technology Officer of Cancog Technologies. GL is the Vice President of Clinical Affairs at Cancog Technologies and served as scientific director of the clinical study. SK is a study coordinator of Vivocore Inc. GL, IM, NM, and SK have no other financial involvement with Nestlé Purina or financial interest in the subject matter and materials discussed in the manuscript.
