# Supplementary material for: Efficacy of a Therapeutic Diet on Dogs With Signs of Cognitive Dysfunction Syndrome (CDS): A Prospective Double Blinded Placebo Controlled Clinical Study
Source: Front Nutr. 2018 Dec 12;5:127. doi: 10.3389/fnut.2018.00127 (PMC6299068; doi:10.3389/fnut.2018.00127)
Supplement: Supplementary file 1 [file Table_1.docx]

Supplementary Table 1: Senior Canine Behavior Questionnaire

Instruction: Indicate your assessment by entering score for each question. The purpose of this questionnaire is to identify behavior changes that have arisen or changed in your pet’s senior years. Therefore please consider your dog’s current behavior compared to when your pet was younger (e.g. <8yrs).

| Scoring Key (severity): **0**=*none (no change)* **1**=*mild* **2**= *moderate* **3**=*severe* | At what age were signs first noticed? | **Score** |  |
| --- | --- | --- | --- |
| **A: Disorientation – Awareness – Spatial orientation (2 questions for inclusion of category)** | | | |
| Gets stuck or has difficulty getting around objects |  |  |  |
| Stares blankly - at walls, floor, or into space |  |  |  |
| Drops food and has problems finding |  |  |  |
| Goes to hinge / wrong side of door |  |  |  |
| Walks into doors / walls |  |  |  |
| Gets lost in home or yard |  |  |  |
| Does not recognize familiar people / familiar pets |  |  |  |
| **B: Altered Social Interactions - People (2 questions for inclusion of category)** | | | |
| Decreased interest or time spent in petting / affection from family members |  |  |  |
| Decreased interest in approaching or greeting family members |  |  |  |
| Spending more time alone or away from family members ___ OR increased following / dependence on family members (“clingy”) ___ |  |  |  |
| More irritable / anxious / fearful / aggressive with family members |  |  |  |
| More irritable / anxious / fearful / aggressive with other family pets  Answer n/a if no other family pets |  |  |  |
| More irritable / anxious / fearful / aggressive with visitors |  |  |  |
| More irritable / anxious / fearful / aggressive with unfamiliar animals |  |  |  |
| **C – Anxiety / response to stimuli** | | | |
| More fearful / anxious / unsettled |  |  |  |
| Increased anxiety when separated from owners (separation distress) |  |  |  |
| More reactive / fearful to visual stimuli (sights) ____ OR  Less reactive / fearful to visual stimuli (sights) ____ |  |  |  |
| More reactive / fearful to auditory stimuli (sounds) ____ OR  Less reactive / fearful to auditory stimulus (sounds) ____ |  |  |  |
| Increased fear of places / locations ___ new environments ___  going outdoors ____ |  |  |  |
| Increased vocalization daytime |  |  |  |
| Increased interest in food / treats ____ OR decreased in food / treats ___ |  |  |  |
| **D: Sleep–wake cycles: (2 questions for inclusion of category)** | | | |
| Waking at night |  |  |  |
| Sleeps less / restless at night ___ delayed falling asleep ___ wakes early___ |  |  |  |
| Walking / pacing at night |  |  |  |
| Night time vocalization |  |  |  |
| Sleeps noticeably more during the day | | | |
| **E: Housetraining, learning and memory (2 questions for inclusion of category)** | | | |
| Indoor soiling urine ___ stools ___ |  |  |  |
| Decrease or loss of signaling to go out |  |  |  |
| Decreased response to learned commands / name / tricks / work |  |  |  |
| Difficulty getting dog’s attention / more distracted / decreased focus |  |  |  |
| Less able or slower to learn new tasks / tricks |  |  |  |
| Reduced ability to adjust to changes |  |  |  |
| **F. Activity (2 questions for inclusion of category)** | | | |
| Increased activity – aimless pacing / wandering |  |  |  |
| Decreased exploration / play with toys |  |  |  |
| Decreased interest in play with family members |  |  |  |
| Decreased interest in play with other pets – Answer n/a if not other pets |  |  |  |
| Decreased interest in spending time outdoors __ walks ___ exercise __ |  |  |  |
| Repetitive behaviors – licking ___ circling ___ chewing ___ star gazing__ |  |  |  |

Supplementary Table 2: Canine Medical Health Questionnaire

Instruction: Please indicate your assessment by entering the number on the scale next to each question. Scoring Key: **0**=*none 1*=*mild 2*= *moderate 3*=*severe*

| Medical Signs  To be completed together with your veterinarian | Age when signs first noted? | **Score** |
| --- | --- | --- |
| Appetite: Increased ___ Decreased ___ |  |  |
| Weight: Weight gain ___ Weight loss ___ |  |  |
| Gastrointestinal: Vomiting ___ Soft stools ___ Constipation ___ |  |  |
| Increased drinking: |  |  |
| Increased urination: More frequent ___ More volume (amount) ___ |  |  |
| Respiratory: Coughing ___ Panting ___ Noisy / labored breathing ___ |  |  |
| Skin problems:  If yes, describe: |  |  |
| Oral: Bad breath ___ Difficulty chewing / swallowing ___ Salivating ___ |  |  |
| Neurological: Shaking / tremors ___ Seizures ___ |  |  |
| Weakness / Incoordination: |  |  |
| Decline in vision: |  |  |
| Decline in hearing: |  |  |
| Altered Mobility: Walking ___ Running ___ Jumping ___ Climbing ___ |  |  |
| Does your pet have any other health concerns not listed above: Y ___ N ___ If yes, describe:  List medications, diet or supplements your pet is taking:  Has the pet been diagnosed as having any other medical problems? Y ___ N ___ If yes, describe: | | |
